# Patterns of funerary variability, diet, and developmental stress in a Celtic population from NE Italy (3^rd^-1^st^ c BC)

**DOI:** 10.1371/journal.pone.0214372

**Published:** 2019-04-17

**Authors:** Zita Laffranchi, Giuliana Cavalieri Manasse, Luciano Salzani, Marco Milella

**Affiliations:** 1 Departamento de Medicina Legal, Toxicología y Antropología Física, Universidad de Granada, Granada, Spain; 2 Soprintendenza per i Beni Archeologici del Veneto, Settore terrirorio, Sede di Padova-Nucleo di Verona, Padova, Italy; 3 Department of Anthropology and Anthropological Museum, Universität Zürich-Irchel, Zürich, Switzerland; Museo delle Civiltà, ITALY

## Abstract

Little is known about the types of social organization characterizing the pre-Roman Celtic populations of Italy. Here, we explore the funerary variability characterizing the late Iron Age site of *Seminario Vescovile* (SV: Verona, Italy, 3^rd^-1^st^ c. BC), and test its possible correlation to diet and relative exposure to developmental stressors. Patterns on funerary treatment (N = 125), δ^13^C and δ^15^N (N = 90), and linear enamel hypoplasia (N = 47) from SV are compared, and their possible association with sex and age-at-death further discussed. Results point to the presence at SV of variable funerary customs while at the same time demonstrating a rather homogenous diet and exposure to developmental stressors: funerary treatment is mainly correlated to age-at-death but do not appear to be associated to either isotopic patterns or hypoplasia frequencies. Accordingly, even if some weak social differentiation may have characterized the individuals buried at SV, this was not reflected in markedly differing living conditions. Our study is the first to attempt an exploration of the links between age, sex, funerary variability, and diet in a pre-Roman Celtic community from Italy. While highlighting the potential of a multifaceted approach in bioarcheology, it also points to a series of analytical and theoretical issues relevant when trying to disentangle the cultural and biological dimensions of social differentiation in the past.

## Introduction

**Fig 1:**
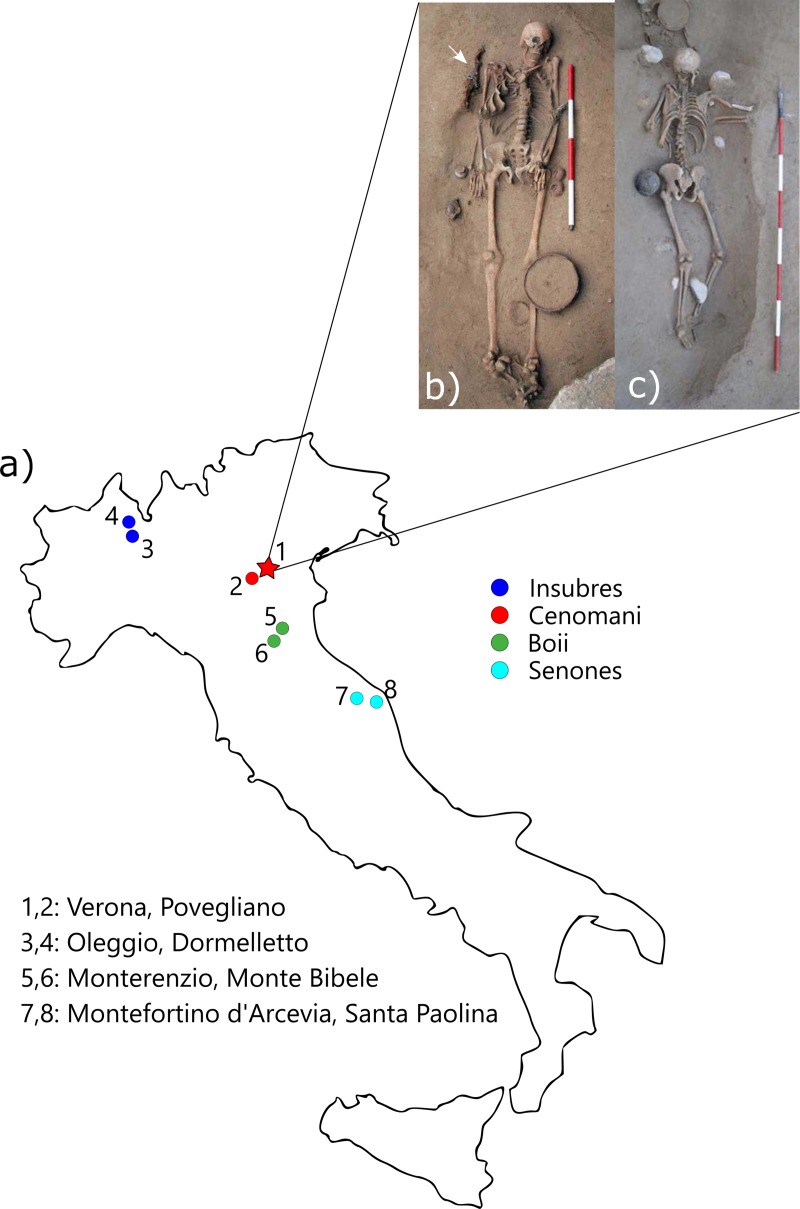
Localization of main Celtic necropolises of Italy and examples of burials from SV. a) The red star identifies the context under study (SV). The colored circles illustrate the location of some of the major Celtic necropolises in Italy mentioned in the text; b) SV: US 2631 (M, MA) supine inhumation accompanied by knife (white arrow). c) SV: US 3207 (F, YA): prone inhumation. Photos by S. Thompson, by courtesy of SABAP-VR Soprintendenza archeologia, belle arti e paesaggio per le province di Verona, Rovigo e Vicenza.

Starting from the 4^th^ century BC the Italian peninsula was involved in the southern spread of populations from Central Europe. This resulted in the development of Celtic settlements in various Northern and Central Italian regions, as documented by both the available written sources (e.g. Livy, Polybius, Tacitus) and a variable amount of archaeological findings including necropolises, isolated objects, and a few architectural structures [1,2]. These data generally reflect the presence of social processes including both examples of cultural admixture with local (greco- and italic) communities, and preservation of transalpine traditions. This scenario is further complicated by the different cultural aspects of the incomers (e.g. Cenomani, Insubres, Boii, Senones), and their variable relationships with the local communities (Fig 1) ([1], see also [3,4]). Some of the main Celtic necropolises investigated in the Italian peninsula are reported in Fig 1A: SeminarioVescovile and Povegliano Veronese (province of Verona) are attributed to the Cenomani [5, 6]. Oleggio and Dormelletto (province of Novara), Monterenzio and Monte Bibele (province of Bologna), and Montefortino d’Arcevia and Santa Paolina di Filottrano (province of Ancona) are attributed respectively to the Insubres, Boii, and Senones [4].

To date, only preliminary hypotheses exist about the type and amount of social organization characterizing these communities. This is particularly relevant given the lack of direct written sources and the possible bias of the available Greco-Roman accounts [3]. Also, generalizing concepts (e.g. the tripartite social organization in common people, military leaders and kings described by Polybius for the Boii) are not necessarily applicable to populations characterized by distinct histories and exposed to variable levels of cultural-(and, at least judging from the epigraphic evidence–genetic) flows ([7, 8, 9]; about the unsuitability of all-fitting concepts for discussions of past cultural groups see [2, 10, 11]).

A proper discussion of the social features characterizing the Italian Celtic communities is further complicated by the paucity of research projects trying to test biocultural hypotheses on the basis of a combined study of archaeological, funerary, and osteological data, and by a regional bias in the available anthropological studies. The bioarchaeological literature on Italian Celtic samples is mostly focused on contexts pertaining to the Emilia Romagna region (Boii Gauls e.g. necropolises of Monterenzio and Monte Bibele), and especially on two research areas: the paleopathological analysis of diet and quality of life [12–16], and the isotopic investigation of mobility patterns [17, 18]. Recent studies by Laffranchi and colleagues [19–21] provide an alternative regional and methodological angle, and include paleopathological and isotopic (diet and infant feeding practices) analyses of skeletal series pertaining to the Cenomani population of North-Eastern Italy. Note however that, with the exception of Sorrentino and colleagues [18], the above studies do not compare different lines of evidence (e.g. biochemical, funerary).

Most studies dealing with the social interpretation of archaeological data from Italian Celtic contexts are focused on the symbolism of hierarchy and militarization among these communities. The presence of swords and other elements of weaponry (e.g. spears, umbo) in male burials, and the isolated presence of horses and chariots in different depositions are consistent with a shared social structure dominated by warrior elites, and a cultural continuity with Celtic traditions from Central Europe [2, 3, 4, 22]. Besides these similarities, however, it appears that these communities tended to differentiate from each other both in the relative frequency of “warrior” depositions and the relative degree of social differentiation. One needs to stress once more the current gap existing between the above reconstructions (invariably based on grave goods data) and the knowledge about the people who practiced and experienced the described customs [1, 3]. This relates, especially, to those layers of the population usually not represented in elite graves, i.e. women, children, and lower status individuals. As such, questions pertaining to the possible relationship (or lack thereof) between *biological* (skeletal and dental data) and *social* (funerary data) identities, and the biological effects of social differentiation among these populations are still almost completely unaddressed.

Here, we try to tackle these issues by exploring funerary features (e.g. type of grave goods, type of burial structure), together with both isotopic patterns (δ^13^C and δ^15^N) and frequencies of linear enamel hypoplasia (LEH) describing possible differential access to food resources and relative exposure to environmental stressors in a scarcely known Italian Celtic community (the Cenomani Gauls).

Enamel hypoplasia is caused by temporary disruptions of ameloblastic activity leading to discontinuities in enamel apposition [23, 24]. Etiological factors include nutritional deficiencies, infectious diseases, congenital anomalies, and trauma [24–33]. Due to its multifactorial etiology LEH is more informative about general levels of physiological stress in a population rather than about the relative incidence of specific disorders. As such, it represent a useful tool for studies aimed at exploring intra- and interpopulation patterns in general life style and living conditions.

δ^13^C and δ^15^N ratios from human collagen are nowadays routinely used for paleodietary reconstruction [34, 35]. In association with other bioarchaeological variables they may be used to explore differential access to food resources stemming from social differentiation [36, 37]. Carbon and Nitrogen isotopic abundances are expressed as δ (delta) values in parts per mil (‰). Accordingly, δ^13^C = (^13^C/^12^C)_sample_ **/ (**^13^C/^12^C)standard− 1) x 1000, and δ^15^N = (^15^N/^14^N)_sample_ **/ (**^15^N/^14^N)standard− 1) x 1000 (see Supplementary Text 1).

Due to their specific photosynthetic pathways, C_3_ and C_4_ plant biomasses differ in their δ^13^C values. C_3_ plants (temperate species, e.g. wheat and barley) are generally enriched in ^12^C and characterized by more negative δ^13^C values (in the range -24/-34‰ V-PDB). C_4_ plants (tropical species including maize, sorghum, millet) on the other hand do not undergo such enrichment in ^12^C and their tissues are therefore characterized by less negative ratios (-6/-19‰ V-PDB) [38, 39]. δ^13^C collagen values from humans and animal samples allow to discriminate between the consumption of terrestrial and aquatic resources [40], but also to analyze the different proportion in the diet of plants characterized by specific photosynthetic pathways (C_3_ vs.C_4_ plants [39, 41]).

δ^15^N values increase about 3–5‰ at each trophic level of the food chain. δ^15^N collagen values can therefore be used to evaluate the position of an animal in the trophic chain, and in consequence the “trophic level” of the human’s diet, permitting estimation of the relative amount of animal and vegetal proteins in the diet [42, 43].

### Biocultural context

Classical sources describe the pre-Roman settlement of Cenomani in the areas corresponding to the actual provinces of Brescia and Verona (Northeast Italy) [4]. This occupation was followed by the gradual incorporation of these areas in the Roman political sphere, a process completed during the 1^st^ Century BC with the foundation of *Brixia* (actual Brescia) and Verona, and in the subsequent acquisition of the status of *Municipium* by the latter in 49 BC [44]. To date, a biological overview of these populations is provided by anthropometric studies [45, 46] and more recent isotopic and paleopathological analyses [19–21, 47–49]. However, the possible social meaning of these data and their link to patterns of social differentiation (age, gender, and status) remain largely unexplored, making the Cenomani one of the most enigmatic people of the Italian Late Iron Age.

The site under study, (*Seminario Vescovile—*SV) was discovered during construction of an underground garage in the Eastern part of Verona and was excavated from 2005 to 2010 (Fig 1 and S1 Fig). Radiocarbon dating of bone collagen indicates its utilization between 3^rd^-1^st^ century BC, whereas typological patterns of grave goods are consistent with its attribution to the Cenomani culture [5, 19, 20, 44,47].

In this study, we decided to enlarge the available radiocarbon data by analyzing a further individual (US 2551, lab ID 2885.1.1). The analysis (performed at the CNA, Sevilla, Spain) points to a ^14^C dating of 2096 ± 32 cal BP (cal BC 199- cal BC 43), further supporting the proposed dating of SV between the 3^rd^-1^st^ c BC (earliest datation: 2169 ± 32 BP, latest datation: 2080±32 BP) [19,20,47]. Among the largest (NMI = 174) and better preserved Celtic necropolises of Italy, SV is characterized by the presence of a complex and heterogeneous funerary behavior. Individuals are mainly laid out in supine position in single graves (only two double burials), with grave goods including animal bones, pottery, and decorations ([5, 47] and Fig 1B). Exceptions to this trend are eleven burials characterized by the unusual position of the body (prone and/or on the sides–Fig 1C), and three inhumations accompanied by animals: a neonate buried with a dog, a male with a ceramic vessel containing the skeletal remains of a canid (domestic dog) fetus, and a female buried with a horse, and further accompanied by a dog skull (near her head) [47]. The funerary complexity of SV is further suggested by a small number of incinerations (n = 7) found in the surrounding (further South-East) of the main cemeterial area and hypothetically associated to the latter [5, 47]. The presence of weapons (absent at SV) in two of these incinerations may suggest that part of the unexcavated area of SV was dedicated to individuals of a different social standing and accordingly provided with a distinct funerary treatment.

Previous anthropological analyses of SV have so far focused on quality of life, dietary patterns, and infant feeding practices [20, 21, 47]. Preliminary paleopathological data (frequencies of enamel hypoplasia and cribra orbitalia) pointed to similar exposures to environmental stressors compared with Roman imperial skeletal series from Italy [47]. Paleodietary results are consistent with a large use of C_4_ plants (supporting the idea of a widespread consumption of millet), and different diets between males and females [20]. The consumption of broomcorn millet (*Panicum miliaceum L*.) and foxtail millet (*Echinochloa crus-galli L*. Beauv. and *Setaria italica* L. Beauv) at SV chime with the results from isotopic studies on Bronze Age samples from the same geographic area [50, 51] This demonstrates the continuation of this specific farming tradition also during the Iron Age, a result of the favorable geological and climatological characteristics of the Po plain [52].

Finally, δ^15^N data of nonadults point to a significant difference of isotopic values around two years of age, a result suggesting a prolonged breast-milk consumption possibly integrated by complementary food sources at least up to this age, similarly to what observed in Roman contexts, and in line with ancient medical sources [53,54].

The aim of the present work is to integrate and expand the results of the above studies, and explore for the first time the possible links between funerary variability, diet, and exposure to environmental stressors in an Italian Celtic community. Specifically, in this study we address the following research questions,

1. Are funerary patterns (type of deposition, type and amount of grave goods) at SV linked to age-at-death and sex?
2. Do funerary patterns at SV correlate with differential access to food resources and exposure to environmental stressors?
3. How variable were the funerary customs at SV?

Results point to: a) a marked influence of age, but not sex, on funerary variability; b) no association between funerary treatment, diet, and exposure to developmental stressors during growth, and c) the presence in the analyzed sample of a funerary variability reflecting a weak social differentiation.

## Material and methods

The complete dataset used in this study is shown in S1 Table. Table 1 shows the distribution of the sample by age and sex.

**Table 1:** Sex and age distribution of the samples used in the study.

	N_F_	N_I_	N_LEH_
NaI	27	14	0
NaII	20	8	0
NaIII	12	9	0
NaIV	7	4	3
YA(F)	11	10	10
YA(M)	20	18	15
MA(F)	12	12	7
MA(M)	16	15	12
Total	125	90	47

N_F_ = funerary information; N_I_ = δ^15^N and δ^13^C information; N_LEH_ = LEH information; NaI: 37–42 weeks; NaII: 0–1 year; NaIII: >1–5 years; NaIV: >5–14 years; YA: 19–34 years; MA: 35–50 years; (F) = females; (M) = males.

The sample includes 125 individuals (corresponding to 72% of the MNI at the site) representing both sexes and different age classes. All individuals (including the youngest ones) were provided with a formal burial. Adult age-at-death was estimated on the basis of the morphological changes of the pubic symphysis, auricular surface of the ilium, and sternal end of the 4^th^ left rib [55–58]. Nonadults’ age-at-death was estimated on the basis of development and eruption of deciduous and permanent dentition, diaphyseal measurements, and degrees of epiphyseal fusion [59–61]. Sex was determined on the basis of cranial and mandibular dimorphic traits, and on the basis of the morphology of pubic symphysis and innominate following standard anthropological methods [62]. The preservation of the dental and skeletal material is overall excellent. Our sample is based on data collected by one of the authors (ZL) during her PhD work which was focused exclusively on the paleopathological and isotopic analysis of the inhumations from SV. Accordingly, the seven incinerations found in the surrounding of the site are not included in this study. Given their possible archaeological relevance, these are in any case discussed in the text.

We subdivided the nonadults individuals in NaI (ca. 37–42 weeks), NaII (0–1 year old), NaIII (>1 to 5 years old), NaIV (>5 to 14 years old). The adults were subdivided in Young Adults (19–34 y) and Middle Adults (MA: 35–50 y). No individuals in the sample were aged between 15–18 years old. Given the small (N = 5) number of individuals possibly aged over 50 years old we decided to include these in the MA age class. Similarly, one female individual aged 17–19 years old was included in the YA class.

### Stable isotope analysis

Isotopic ratios for the population of SV have been previously presented elsewhere [20, 21]. We refer the reader to those works and to S1 Text for a detailed description of the standards and analytical protocols used in this study.

Bone collagen was extracted from 91 human ribs and 7 animal bones (1 goat/sheep, 2 cows, 2 horses and 2 dogs) following the protocol described by Bocherens et al. [63, 64]. Analysis of nitrogen and carbon ratios was carried out by means of a Carlo Elba NC1500 (Milan, Italy) elemental analyzer on line with a Delta Plus XP (ThermoQuest, Bremen, Germany) mass spectrometer (EA-IRMS). Commercial CO_2_ and N_2_ were used as the internal standards for the carbon and nitrogen isotopic analyses (S1 File).

### LEH

Presence of LEH was observed on permanent upper and lower incisors and canines with the aid of a magnifying glass and scored following the criteria of Steckel and colleagues [65]. We considered only teeth presenting ca 90% of the crown, and lacking extensive occlusal wear (i.e. a maximum wear stage of 6 of Smith’s system [66]). These data were then used to obtain individual LEH binary scores. For this purpose, only individuals presenting at least 7 out of 12 teeth were considered, and LEH was assessed as present if hypoplastic defects were visible on at least one tooth.

### Funerary data

Funerary data from SV were analyzed following the protocol of Milella and colleagues [67]: each burial was coded according to ten binary (presence-absence) variables describing various funerary aspects (type of grave goods, orientation and position of the individual, presence of animal offerings and burial structure–S2 Table). The number of variables was set in order to maximize the sample size, minimize the missing data, and provide a general characterization of burial variability. Recognizing the possible limits of binary coding for archaeological data [68], we also recorded the number of items (ceramics, rings, and pins) found in each burial. Note that, as mentioned, all the analyzed individuals were provided with a formal burial. Accordingly, we can exclude any possible bias to our analyses due to the presence of commingled remains.

### Statistical analyses

Statistical analyses were subdivided according to the following steps:

#### Age and sex

In order to check for differences in presence of funerary features between age classes and sexes we performed Fisher’s exact tests on the contingency tables of age (classes) and sexes by variable (presence-absence).

The influence of age and sex on number of rings, ceramics, and pins was tested by using a Poisson regression, with the count of each item as dependent variable and age class, sex, and their interaction as predictors. In this and all the other models (see below) age was treated as a categorical variable. In case of significant effects of one or more of the predictors, we performed a post hoc test comparing each group’s estimated marginal means by a z test.

Note that in this and all the subsequent regressions (see points 2 and 3), the interaction between sex and age was dropped from the model if preliminarily found to be not significant.

#### LEH

We checked for the possible association between funerary features and presence of LEH. Since only 3 nonadults had observable permanent dentition we narrowed the analysis to the adult sample. In order to control for the possible bias of age (i.e. dental wear) and sex on LEH frequencies we preliminarily performed a logistic regression of LEH vs. sex, age, and their interaction as predictors. On the basis of the results, we then decided to either subdivide or group age classes and sex when comparing absence-presence of funerary features for absence-presence of LEH by means of a Fisher test. This statistical approach was preferred to a logistic regression of LEH vs. age, sex and funerary features given the risk of overfitting of the model (11 predictors vs. 44 individuals). Note that in this and the subsequent analyses of the adult sample the variable “bead/pendants” was not considered given its total absence in this part of the burials of SV.

#### Stable isotopes

We analyzed the possible association between isotopic values and funerary treatment. Since previous works [21, 20] confirmed at SV the expected higher δ^15^N values associated to breastfeeding, and pointed to different isotopic values between sexes, we first needed to control for these factors. For δ^15^N we repeated all analyses in adults and nonadults. In order to decide if to group age classes of each set we then performed a general linear regression including age, sex, and their interaction as predictor (only adults were tested for the latter two variables), and δ^15^N as the dependent variable. For simplicity, the same approach was followed when analyzing δ^13^C. We then used a Wilcoxon test to check for differences in isotopic values between presence/absence of funerary features.

#### Funerary variability

Multivariate patterns in the funerary dataset at SV were examined in order to test for the presence of discrete funerary clusters. We first checked for multicollinearity by testing the association of absence/presence between each variable by means of a Fisher’s test. Variables showing a significant association were then dropped from the following analyses.

Multivariate differences between age classes and sexes were tested using a permutational multivariate analysis of variance (PERMANOVA, 10.000 permutations) on the dataset of funerary variables after converting it to a distance matrix using an asymmetric binary distance. The latter is especially suitable in cases, like the present one, where variables refer to the presence of rare features resulting in large frequencies of shared absence (0) between individuals. PERMANOVA is a method closely related to the analysis of variance, allowing the test for the presence of differences/ structures in a dataset represented by a distance matrix, and given a set of independent variables (in our case age classes and sex). This procedure was performed on the full sample (with only age as independent variable), and separately on adults (with age, sex, and their interaction as independent variables). Significant results were further explored with post hoc pairwise comparisons.

PERMANOVA was coupled with a hierarchical cluster analysis (UPGMA method) of the binary funerary dataset converted to a (asymmetric) distance matrix, using the *pvclust* function of the R package pvclust [69]. The significance of the resulting clusters was then checked by means of multiscale bootstrapping (10.000 replicates), and expressed in percentage as AU (approximately unbiased) p values. Clusters with AU ≥ 95% were considered to be strongly supported by the data. Differences between clusters in sex, age composition, and LEH frequency were analyzed with pairwise Fisher tests, while patterns of isotopic values across clusters by means of pairwise Wilcoxon tests.

Finally, we plotted the geographic coordinates of each burial and visually inspected the presence of possible spatial patterns at SV related to age, sex, funerary clusters, and/or isotopic values. Lines representing Kernel density estimates were used to better define possible differences in density between burials pertaining to contrasting funerary clusters or sexes.

In all tests, alpha was set at 0.05, and a Bonferroni correction was applied in all cases involving multiple testing.

Analyses were performed in R version 3.5.0 [70]. Pairwise Fisher and Wilcoxon tests were computed respectively with the functions *pairwise*.*fisher*.*test* of the package “reporttools” [71] and *pairwise*.*wilcox*.*test* (package “stats”). Estimated marginal means of the Poisson models and post hoc z test were obtained, respectively, with the functions *marginal* and *pairs* of the package “emmeans” [72]. PERMANOVA and related post hoc tests were computed respectively using the functions *adonis* of the package “vegan” [73] and *pairwise*.*adonis* (package “pairwiseAdonis” [74]). Cluster analysis was performed with the function *pvclust* of the package “pvclust” [69]. Generalized linear models were performed with the function *glm* (package “stats”). Plots were made in R and JMP 13.0.0 (SAS Institute Inc., Cary, NC, 1989–2007) and edited with Inkscape 0.92 (**www.inkscape.org**). Kernel density estimates were computed with the function *stat_density_2d* of the R package “ggplot2” [75].

## Results

### 1) Age and sex vs. funerary features

#### Age vs. presence of funerary variables

**Fig 2:**
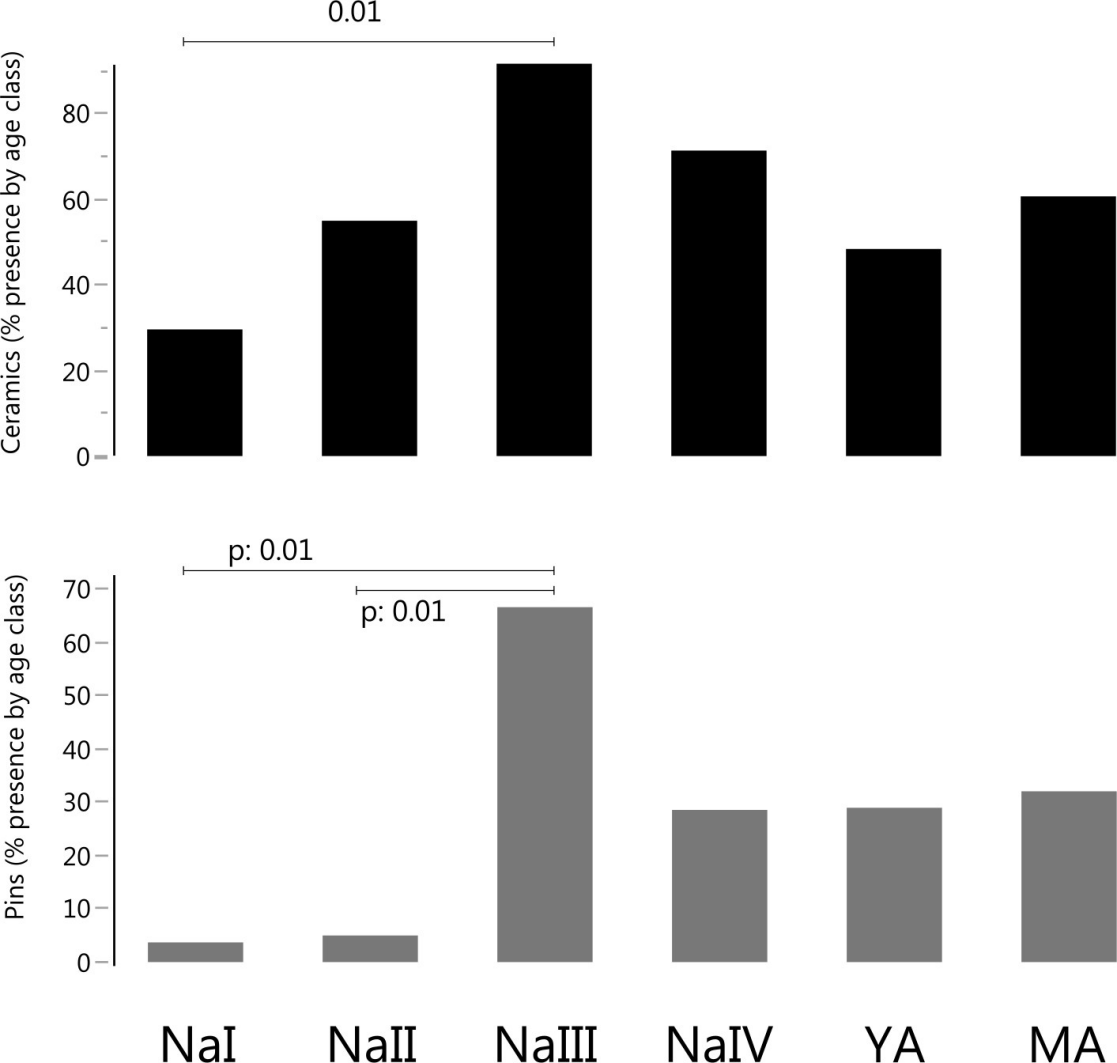
Frequency of presence of ceramics and pins by age class, and Bonferroni-adjusted p values of significant differences between classes.

A comparison of funerary features across age classes (without separating adult sexes) highlights a significantly higher frequency of ceramics in NaIII compared with NaI, and of pins when comparing NaIII to both NaI and NaII (Fig 2, Table 2). As already mentioned (see above) due to the total absence of beads/pendants in burials of adult individuals, this variable was not considered when analyzing YA and MA age classes.

**Table 2:** Absolute (n) and relative (%) frequencies of individuals characterized by the presence of each funerary feature per age class, and Bonferroni-adjusted results of pairwise Fisher’s tests. N: sample size; asterisks indicate pairwise comparisons yielding significant results. *: p = 0.01; ^**•**^: p = 0.01; ^**••**^: p = 0.01.

	NaI (N:27)		NaII (N:20)		NaIII (N:12)		NaIV (N:7)		YA (N:31)		MA (N:28)	
	n	%	n	%	n	%	n	%	n	%	n	%
Rings	0	0.0	2	10.0	0	0.0	2	28.6	4	12.9	7	25.0
**Ceramics**	**8**	29.63^*^	11	55	**11**	91.7^*^	5	71.4	15	48.4	17	60.7
**Pins**	**1**	**3.7**^**•**^	**1**	**5**^**••**^	**8**	**66.67**^**•,••**^	2	28.6	9	29.0	9	32.1
Non supine	7	25.9	2	10.0	0	0.0	1	14.3	6	19.4	4	14.3
Structure	9	33.3	6	30.0	1	8.3	0	0.0	4	12.9	4	14.3
Orientation: EW	5	18.5	1	5.0	0	0.0	0	0.0	1	3.2	1	3.6
Animal offerings	1	3.7	3	15.0	0	0.0	1	14.3	4	12.9	5	17.9
Coin	0	0.0	2	10.0	3	25.0	1	14.3	3	9.7	6	21.4
Knife	0	0.0	0	0.0	1	8.3	0	0.0	3	9.7	2	7.1
Beads/pendants	2	7.4	2	10.0	2	16.7	0	0.0	0	0.0	0	0.0

#### Sex vs. presence of funerary variables

No difference was found in frequency of funerary features between sexes (Table 3). Also, no funerary differences were found when comparing different age classes in each sex (data not shown).

**Table 3:** Absolute (n) and relative (%) frequencies of individuals characterized by the presence of each funerary feature by age class and sex. Bonferroni-adjusted results of pairwise Fisher’s tests are not reported due to the lack of significant results.

	YA F (N:11)		MA F (N:12)		YA M (N:20)		MA M (N:16)	
	n	%	n	%	n	%	n	%
Rings	4	36.4	3.0	25.0	0	0.0	4	25.0
Ceramics	5	45.5	7.0	58.3	10	50.0	10	62.5
Pins	3	27.3	4.0	33.3	6	30.0	5	31.3
Non supine	3	27.3	2.0	16.7	3	15.0	2	12.5
Structure	1	9.1	3.0	25.0	3	15.0	1	6.3
NS	0	0.0	1.0	8.3	1	5.0	0	0.0
Animal offer	1	9.1	2.0	16.7	3	15.0	3	18.8
Coin	0	0.0	3.0	25.0	3	15.0	3	18.8
Knife	0	0.0	0.0	0.0	3	15.0	2	12.5
Beads/pendants	0	0.0	0	0.0	0	0.0	0	0.0

#### Age vs. number of rings, ceramics, and pins

S3 Table shows the summary statistics of the number of rings, ceramics, and pins by age class and sex. The Poisson models highlight a significant effect of age on the number of pins and ceramics (Table 4). A post hoc test between age classes reveals however only three significant differences, all related to the number of pins (NaIII versus NaI, p: 0.0062; NaIII vs. NaII, p: 0.00256; NaIII vs. MA, p: 0.0453). In all cases NaIII is the age class with more items.

**Table 4:** Coefficients of the Poisson regression of number of rings, ceramics, and pins on age and sex for the full dataset (with NaI set as reference level in the model), and adults (with YA and F set as reference levels in the model). N = number of items; ns = not significant.

	Estimate	SE	z	p
**N Rings (Full dataset)**				
Intercept	-19.300	1814.000	-0.011	ns
Age: NaII	17.410	1814.000	0.01	ns
Age: NaIII	0.000	3271.000	0	ns
Age: NaIV	18.050	1814.000	0.01	ns
Age: YA	17.660	1814.000	0.01	ns
Age: MA	18.460	1814.000	0.01	ns
**N Ceramics (Full dataset)**				
Intercept	-0.731	0.277	-2.635	0.008
Age: NaII	0.731	0.356	2.052	0.040
Age: NaIII	1.935	0.319	6.061	<0.001
Age: NaIV	0.864	0.449	1.924	ns
Age: YA	0.763	0.329	2.319	0.020
Age: MA	1.350	0.310	4.353	<0.001
**N Pins (Full dataset)**				
Intercept	-2.603	0.707	-3.681	<0.001
Age: NaII	0.300	1.000	0.3	ns
Age: NaIII	2.683	0.760	3.532	<0.001
Age: NaIV	1.350	1.000	1.35	ns
Age: YA	1.654	0.764	2.165	0.030
Age: MA	1.350	0.791	1.708	ns
**N Rings (Adults)**				
Intercept	-1.183	0.460	-2.571	0.010
Sex: M	-0.845	0.485	-1.744	ns
Age: MA	0.731	0.501	1.457	ns
**N Ceramics (Adults)**				
Intercept	-0.204	0.244	-0.837	ns
Sex: M	0.346	0.234	1.475	ns
Age: MA	0.612	0.225	2.716	0.007
**N Pins (Adults**				
Intercept	-0.907	0.408	-2.223	0.026
Sex: M	-0.066	0.458	-0.144	ns
Age: MA	-0.309	0.458	-0.674	ns

#### Adults: age, sex vs. number of rings, ceramics, and pins

When considering only adults, we first excluded from all three models the interaction between age and sex due to lack of significance (p values for the models of number of rings, ceramics, and pins: respectively 0.993, 0.151, and 0.682). Age is significantly associated to the number of ceramics (Table 4), and the positive coefficient of this predictor points to an increase in the number of items from YA to MA. This is further supported by the post hoc test, which shows a difference between the two age classes in each sex, and between YA females and MA males (S3 Table).

### 2) LEH vs. age, sex, and funerary features

The logistic regression performed without the interaction between sex and age due to its lack of significance (p: 0.673) highlights age as a significant predictor of LEH (Table 5). Accordingly, Fisher’s tests between LEH and funerary variables were performed separately for the two adult age classes, but grouping the sexes in order to maximize sample sizes. The analysis failed to reveal any association between hypoplastic defects and type of funerary treatment (Table 6).

**Table 5:** Adult sample: Coefficients of the logistic regression of LEH vs. Sex and Age (F and YA are set as reference levels in the model).

	estimate	SE	z	p
Intercept	1.2005	0.6226	1.928	ns
Sex: M	-0.707	0.6712	-1.053	ns
Age: MA	-1.3069	0.6506	-2.009	0.0446

**Table 6:** Adult sample: absolute (n) and relative (%: row percentages) frequencies of LEH absence and presence by absence and presence of funerary variables. All Bonferroni-adjusted results of Fisher’s tests by age class are not significant and not reported.

		YA		MA	
		Absent	Present	Absent	Present
		n (%)	n (%)	n (%)	n (%)
Rings	Absent	8 (38.1)	13 (61.9)	9 (64.3)	5 (35.7)
	Present	0	4 (100)	3 (60)	2 (40)
Ceramics	Absent	2 (20)	8 (80)	3 (50)	3 (50)
	Present	6 (40)	9 (60)	9 (69.2)	4 (30.8)
Pins	Absent	5 (29.4)	5 (70.6)	8 (66.7)	4 (33.3)
	Present	3 (37.5)	5 (62.5)	4 (57.1)	3 (42.9)
Non supine	Absent	8 (40)	12 (60)	12 (75)	4 (25)
	Present	0	5 (100)	0	3 (100)
Structure	Absent	6 (28.6)	15 (71.4)	10 (58.8)	7 (41.2)
	Present	2 (50)	2 (50)	2 (100)	0
Orientation: EW	Absent	8 (32)	17 (68)	11 (61.1)	7 (38.9)
	Present	0	0	1 (100)	0
Animals	Absent	5 (23.8)	16 (76.2)	10 (58.8)	7 (41.2)
	Present	3 (75)	1 (25)	2 (100)	0
Coins	Absent	6 (26.1)	17 (73.9)	9 (60)	6 (40)
	Present	2 (100)	0	3 (75)	1 (25)
Knives	Absent	5 (22.7)	17 (77.3)	11 (61.1)	7 (38.9)
	Present	3 (100)	0	1 (100)	0

### 3) Isotopic values vs. funerary patterns

In nonadults, neither age nor funerary variables appear to predict δ^15^N or δ^13^C values (S4 Table; S5 Table). We dropped the interaction between sex and age from both models for adult isotopic values after checking its lack of significance (for δ^15^N or δ^13^C p respectively = 0.52 and 0.36).

In adults, both age and sex show a significant effect on δ^15^N values (S4 Table). When controlling for these variables, no association is detected between funerary treatment and isotopic values (S5 Table).

Sex, but not age, is confirmed as a significant predictor of δ^13^C values (S4 Table). Accordingly, the association between isotopic values and funerary treatment is investigated in this case by performing a Wilcoxon test between sexes, but without subdividing the sample in age classes. No isotopic differences were found associated to funerary variables (S5 Table).

### 4) PERMANOVA and cluster analysis of funerary features

Results of the Fisher tests on the complete dataset suggested excluding, due to significant collinearity, the absence/presence of ceramics and coins from the following multivariate analyses (results not shown). Conversely, no collinearity was identified among adults, and all funerary variables were accordingly used when analyzing this sample.

The PERMANOVA on the full set show that age significantly influences funerary variability (Table 7). Significant pairwise differences are between the classes NaI–NaIII (p: 0.015), and NaII-NaIII (p: 0.03). Table 2 shows that the biggest differences in the first case relate to the relative frequencies of non-supine individuals, funerary structures, EW oriented burials (higher in NaI), and of pins and beads/pendants (higher in NaIII). In the second case, the largest differences relate to the frequencies of rings, non-supine individuals, and animal offerings (higher in NaII), and of pins and knives in NaIII. PERMANOVA on the adult sample does not show any effect of either sex or age on multivariate funerary variability (Table 7).

**Table 7:** Results of PERMANOVA on the full dataset, and adults.

	Df	Sums Of Sqs	Mean Sqs	F.Model	R2	p
**Full dataset**						
Age	5	4.063	0.8127	2.1729	0.0837	0.0021
Residuals	119	44.507	0.3740		0.916	
Total	124	48.571			1	
**Adults**						
Sex	1	0.20262211	0.2026	0.5462	0.0096	ns
Age	1	0.09182006	0.0918	0.2475	0.0044	ns
Residuals	56	20.7743374	0.3710		0.986	
Total	58	21.0687795			1	

**Fig 3:**
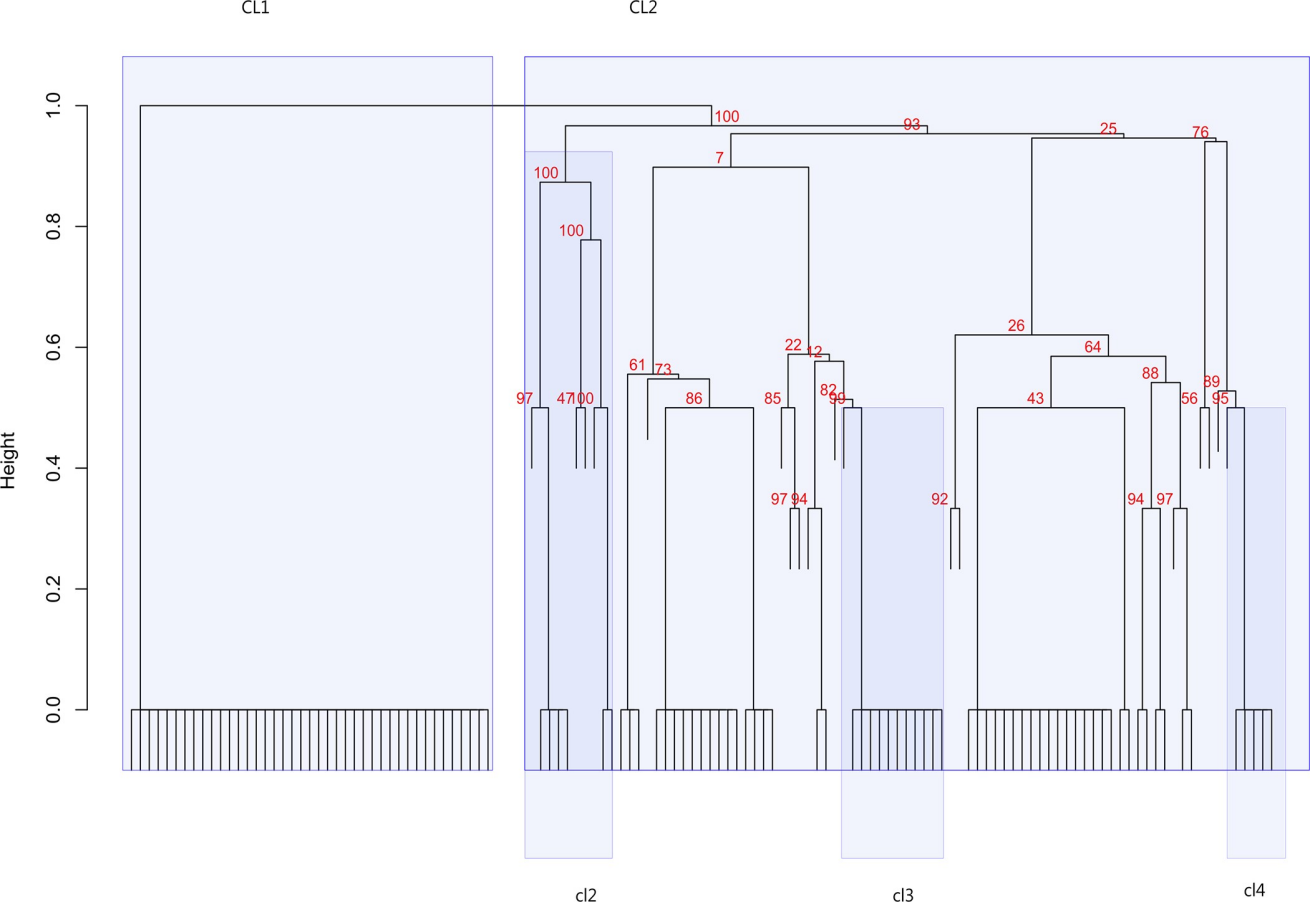
Result of UPGMA cluster analysis of the binary funerary dataset.

Results of cluster analysis point to a partition of the dataset in two main groups (henceforth *CL1* and *CL2*). Using as threshold a sample size ≥5, three smaller clusters (henceforth *cl2*, *cl3*, and *cl4*) are identified in CL2 (Fig 3).

The significance of clusters is checked by means of multiscale bootstrapping (10.000 replicates), and expressed in percentage as AU (approximately unbiased) p values. Clusters with AU ≥ 95% are considered to be strongly supported by the data. Only significant clusters including at least 5 burials are discussed in the text and are highlighted by rectangles.

We checked for the possible association between the above clustering, sex and/or age performing two different comparisons: a) CL1 versus CL2, and b) CL1 vs. the smaller clusters, plus individuals from CL2 not included in any group (henceforth *NC2*). The only significant result is a difference in age composition between cl3 (mostly composed by NaI and NaII individuals, and NC2 (more homogenous and with higher frequencies of adults) (S6 Table).

In terms of funerary variables (S7 Table, S2 Fig), the clearest difference is between CL1 and CL2, with the second cluster presenting a significant higher frequency of rings, pins, coins, animal remains, burial structures, and non-supine inhumations (S7 Table). CL1 and the smaller clusters differ for all variables, with the exception of the frequency of ceramics, knives, and beads/pendants (S7 Table). Among results of the pairwise comparisons, interesting are higher frequencies of funerary structures in cl3, of animal offerings in cl4, and of EW orientation in cl2 (S7 Table).

No significant differences in isotopic values or LEH frequency were observed between CL1 and CL2 (data not shown). A comparison using the smaller clusters was not performed due to the excessively small sample sizes.

A visual inspection of the spatial distribution of burials (S3 and S4 Figs) does not allow to identify spatial patterns on the basis of age, funerary treatment (main clusters), or isotopic values with the exception of a slight tendency for males to occupy the South-Western area of the necropolis.

## Discussion

### 1) Age and sex differences

#### Age

Though results point to an increase of frequency of grave goods (and also number of objects) with age, this pattern is significant only among nonadults for ceramics and pins. The sudden increase of grave goods presence in the class NaIII points to a social change taking place between 1–5 years old, possibly related to a stronger incorporation of the individual in the family unit and community (cf. [76–78], and [79] for a similar pattern during the Final Bronze Age in Italy). The lack of differences between nonadults and adults would suggest either that the passage into adulthood did not correspond to a marked change of funerary treatment, or that the latter was actually present, but undetected due to the limits of our analytical approach (see below). Additionally, we must consider that, if social status at SV was at least in part inherited at birth (e.g. membership to kinship group–see below), this may have resulted in a funerary similarity between nonadults and adults, therefore further obscuring possible age-related patterns.

It is also important to note that skeletal, chronological, and social ages do not necessarily overlap [77, 80–82]. The actual chronological age of an individual could indeed deviate from that estimated from skeletal and dental changes. Also, changes in social identity linked to age transitions are culturally variable, essentially fluid, and entwined with additional variables (e.g. gender, status). As such, binary concepts (e.g. “childhood” vs. “adulthood”) familiar to a modern Western perspective, may only partially apply to other (including past) cultures [80]. Also, our analyses are performed on fixed age classes (ordinal variable). This may hamper a full understanding of the social changes linked to growth and ageing (which are continuous processes).

It is interesting to note that variables describing the position of the individual (supine/non-supine), and type and orientation of burial (with or without structure, oriented N-S or E-W) do not show any pattern by age. This suggests a lack of relation to specific social differences (vertical and/or horizontal) for these features, and, possibly, their link to general funerary norms shared by the whole community.

#### Sex

An unexpected result is the lack of funerary differences between sexes. The absence at SV of those “gendered” items (e.g. *torques* and weapons) found in other Italian Celtic contexts [3,4,22,83–85] is at least in part responsible of this homogeneity. Note however that: a) in these contexts the frequency of weapons is actually quite variable [22], and b) their absence at SV could be due to the partial excavation of the necropolis. This is supported by an earlier unpublished finding (in 2001) of a cremation with a sword as grave good, and the subsequent discovery about 120 m South-East of SV [5, 47] of 6 cremations, one presenting a sword and an umbo (the central element of the shield; the remaining five cremations did not present weapons, and were not characterized by specific types or amount of items) [5, 47]. These findings hint at the possibility of part of the unexcavated area being dedicated to individuals of higher status characterized by a different funerary treatment (cremation, deposition of weapons). Even if this is at the moment only a working hypothesis (the association between these area and SV is yet to be definitely proven). It is clear therefore that we must consider our results with caution due to the possibility of SV representing only a section (possibly composed by lower status individuals) of the original community.

### 2) Isotopic and LEH correlations

#### Stable isotopes

Considering stable isotopes, the clearest result is the effect of sex on both δ^15^N and δ^13^C values, confirming therefore the observations of Laffranchi and colleagues [20], who evidenced higher δ^15^N values (p < 0.01) and more negative δ^13^C values (p: 0.008) in males. These patterns are interesting when compared with the large overlap of males and females in funerary features. Specifically, besides confirming the difficulty of reconstructing gender identities from the archaeological record (see [86, 87]), our results suggest that social differences between sexes at SV were mainly expressed in terms of lifestyle (differential access to food sources) rather than symbolically by means of specific funerary features. When controlling for sex, values of δ^15^N do not show any correlation with funerary treatment. This overlaps with previous research from both Continental and Insular Celtic contexts [88–90] while at the same contrasting with others [36, 37, 91–93]. In particular, δ^15^N data point to a homogenous access to animal proteins and fits with the absence at SV of markedly rich burials. Together with the lack of spatial patterns for both isotopic values, these results may reflect the absence of well-defined social differences between the individuals buried at SV. In previous studies [35, 37, 91–93], differences in δ^15^N were mostly found when comparing burials with weapons to the rest of the population, and were related to higher protein intake of a specific social group (male warriors). SV does not include individuals definable as warriors or of high status: this would explain the low variability of both funerary features and δ^15^N values. Also, our data do not allow for differentiation between specific (and possibly unequally accessible) sources of dietary proteins (e.g. different cuts of meat, different animals, meat vs. dairy foods—cf. [88]).

Similarly to δ^15^N, also δ^13^C values do not differ between contrasting funerary treatments, a result that further supports the hypothesis of the only social difference in diet at SV being the one between sexes.

#### LEH

The relative frequency of LEH at SV (presence in adults: 54% of 44 individuals) is higher than those described for the Boii Gauls from Casalecchio di Reno and the Etruscan population of Spina (respectively 31.3% and 31.2%) [15, 94]. This may suggest for the Cenomani a relatively higher exposure to stress episodes during growth when compared with contemporary populations of Northern Italy. Also, in both Boii and Etruscans LEH frequencies differ between males and females sexes (with higher frequencies among males) [15, 94]. The lack in our study of similar differences suggests that, while males and females at SV were apparently characterized by different diets (see above), during growth they experienced in average similar levels of environmental stressors. Alternatively, given the higher physiological buffering characterizing females [95], it is possible that even if women were exposed during their childhood to higher levels of stressors, the intensity of the latter was not enough to produce significantly higher rates of disruption of their ameloblastic activity.

The absence of association between LEH and funerary treatment align with those on δ^15^N (and funerary, see below), which depict a population lacking marked social differentiations. Note however that some caution is necessary when using LEH in biocultural reconstructions. A possible issue is indeed the variability of the population under study about individual predisposition toward developing enamel defects. Dental development is influenced by the interplay between genetic, epigenetic, and environmental factors [96], and a genetic predisposition to enamel defects has been found [97]. Therefore it is possible that our inability to observe an association between LEH and specific funerary treatment may be at least in part due to the complex etiology of this variable.

### 3) Funerary variability

Our last research question addresses the type and degree of variability characterizing funerary customs at SV. As already mentioned, the social organization of the Cenomani communities is almost completely unknown. This, while making the present study the first attempt to explore this topic from a bioarchaeological perspective, inevitably complicates the interpretation of our data. Keeping in mind these caveat, some results are however interesting for their possible cultural significance.

**Fig 4:**
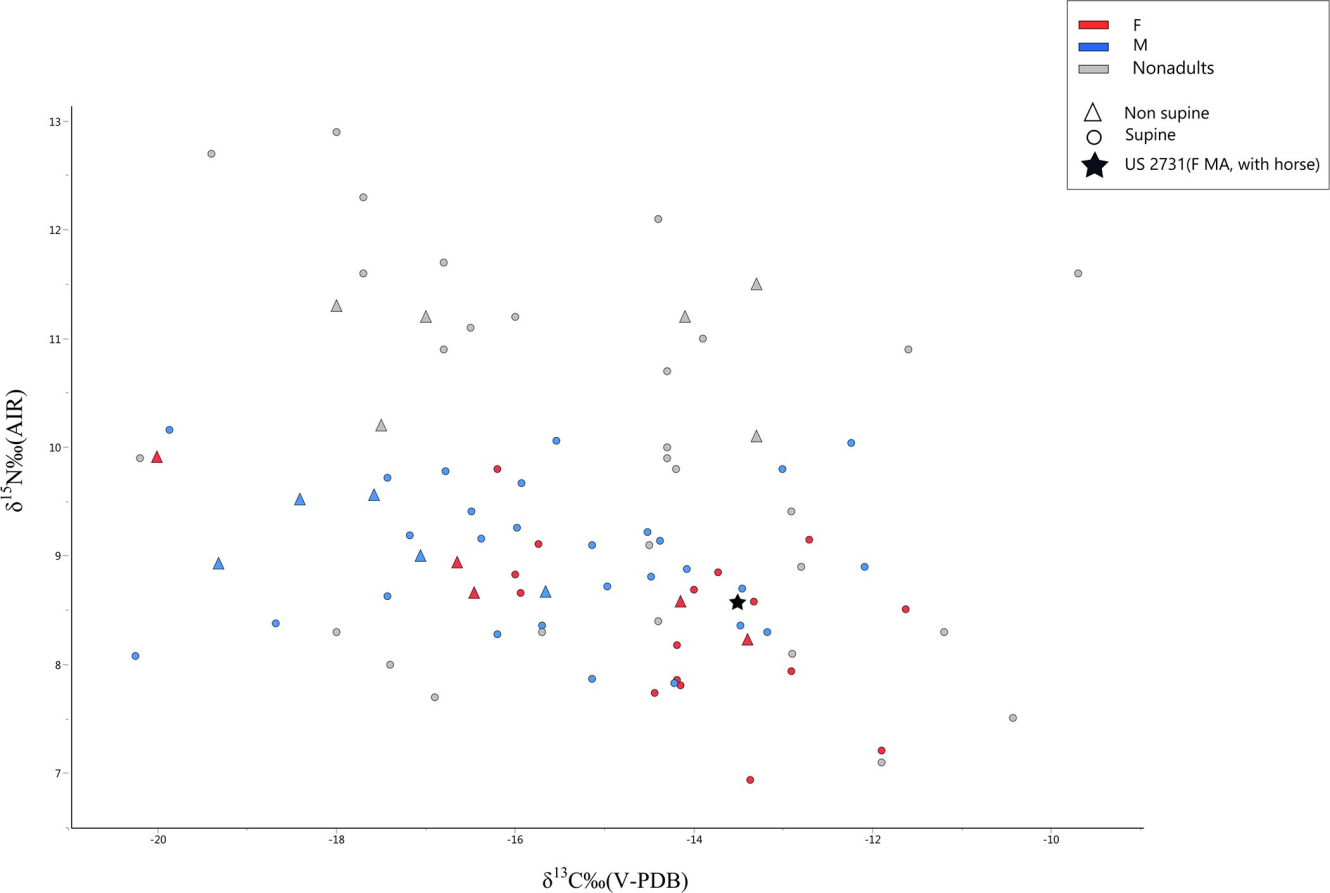
Plot of δ^15^N vs. δ^13^C values. Colors indicate sexes. Triangles: non-supine burials, Star: female burial with horse.

First, if we accept that the two incinerations with weaponry and SV belong to the same funerary context, this would fit with similar findings (e.g. Povegliano Veronese [6], Santa Maria di Zevio [98]), suggesting also for this population the presence of a society featuring an elite composed (at least in part) by warriors. This type of vertical social differentiation would fit not only with what previously postulated for the Cenomani [6, 98–100], but also for other Celtic groups in Italy [3, 17, 22, 101], and, in general, Continental Europe [36, 37, 91, 93]. More specifically, it hints at the possibility that the individuals buried at SV represent a “non-elitarian” segment of the original population. Even accepting this hypothesis however (and at present we can only consider it as a possibility), it is important to stress that the burials of SV *do* present some degree of funerary heterogeneity, as demonstrated by the results of the cluster analysis. This raises the question of the possible social relevance of this ritual variability. The absence of age or sex differences between main funerary clusters excludes their association to these factors. Similarly, the lack of any trend in both isotopic and LEH frequencies militates against possible differences in diet and/or exposure to stressors between the individuals included in each group. This is exemplified by US 2731, a middle adult female buried with a horse lying on the top of her and presenting as grave goods two bronze rings. This finding, unique at SV, reminds of cases from other Italian Celtic contexts involving the deposition of equid skeletons either isolated or in association with human burials. Horses appear therefore to have been defined by a specific symbolic meaning among the La Tène populations of Italy, possibly as sign of social standing, and not necessarily linked to the status of warrior. Besides US 2731, in two other cases from Italy (Senones necropolis of Montefortino di Arcevia—Ancona, and Cenomani necropolis of Carzaghetto–Mantova), a horse was associated to the burial of a female individual [102]. It is therefore possible that US 2731 held a special social standing in her community. However, her isotopic signatures do not deviate from those of the rest of the population (Fig 4). Similarly, the lack of LEH on the few (n = 4) observable teeth cannot be considered anomalous at SV, given the overall low frequency of enamel defects in this sample. It seems therefore that, despite her different funerary treatment, this woman was not characterized by a specific diet nor by a markedly better (or poorer) environment during growth.

A further interesting finding at SV is the subset (n = 20, 16% of the analyzed sample) of burials featuring a non-supine position of the individual. Similarly to the Roman times ([67] and references therein), the possible meaning of a differential handling of the dead during the Iron Ages is unclear [91, 103]. A recent study of burials dating to the German Early Iron Ages [91] concluded, on the basis of paleopathological and isotopic data, that individuals “informally” buried (e.g. prone) were part of the lower segment of a highly hierarchal society. Our analyses failed to identify significant differences in isotopic values or LEH frequencies between supine and non-supine individuals (Fig 4, Table 6, S5 Table).

Also, tests for collinearity in the funerary dataset (see Results section 4) do not show any association between the position of the individual and other variables. Taken together, these results reject the hypothesis of non-supine depositions at SV being the result of a “lower” social standing. Rather, they may be either devoid of any specific symbolic meaning, or (more likely) they may be linked to other factors (e.g. mobility, kinship, chronology—see below) unexplored in this study.

The smaller clusters (cl2, cl3, cl4) are interesting, since they demonstrate the presence at SV of a mortuary heterogeneity transcending the basic absence or presence of grave goods (i.e. CL1 vs CL2). A discussion of these funerary subgroups is however hampered by their small size, which makes their statistical analysis meaningless. As mentioned, it is possible that at least part of the funerary variability observed at SV is linked to factors not necessarily related to vertical social differences (e.g. different geographical origin, kinship groups, and different chronology of burials). A relationship between funerary and mobility patterns seems unlikely on the basis of preliminary data on Oxygen stable isotopes (δ^18^O, in preparation) which point to a population composed mostly by local individuals. The presence of kinship groups at SV is an interesting hypothesis that however cannot be tested at the moment in the absence of further data (paleogenetic and/or phenetic). In any case, the results of our analyses allow concluding that, even if some form of social differentiation characterized the section of the population analyzed in this study, this was probably not really pronounced. This would fit with the archaeological data from other Celtic communities in Italy [1, 4, 22] and Central Europe [37, 91, 104]. Moreover, during the final phases of Iron Age there is a change in burial rites consisting with the dismissal of cremations and richly furnished burials, and the passage to a system of flat inhumations provided with standardized and less rich items (besides the occasional deposition of weaponry) [36, 37, 91, 104]. This trend, consistent with an increasing social homogenization [36, 37, 104], may also partly explain the funerary customs observed at SV, which indeed feature quite a nuanced variability. If funerary features at SV are indeed influenced by similar diachronic changes in social organization, it is possible that the incinerations found in the surrounding of SV relate to a different phase of use of the necropolis. Similarly, the funerary clusters highlighted by our multivariate analysis may be the by-product of chronological differences between burials. Only an extended ^14^C datation program and the finalization of the typological study of grave goods will allow testing these interesting hypotheses.

### 4) Limitations and future directions

A number of issues must be taken into account while evaluating our results. These include both methodological and theoretical problems.

Fist, the general relevance of our analyses is strongly limited by the fact that our sample likely represents just a portion of the original necropolis. This issue, while relevant, is however typical, and unavoidable when dealing with excavations like the one carried at SV (rescue archaeological works). Similarly, a comparison with other Celtic contexts from Italy must be carried with caution given 1) a shared poor knowledge about the social aspects of Celtic Italian communities, and 2) the risk of assuming in such comparisons a supposed cultural continuity between these groups (e.g. Boii, Senones, and Cenomani).

Second, our funerary variables are few, and likely to provide only a rough description of each burial. It is possible that our methodology led to an underestimation of the real funerary variability at the site. Variables in this study were however chosen in order to maximize the sample size and comparisons between individuals. Only after the completion of the typological analyses at SV (in progress: [105–108]) it will be possible to perform a more detailed subdivision of grave good classes. Besides, it is important to stress that the use of funerary data for the reconstruction of past social organization is characterized by both advantages and theoretical issues [109–117]. The symbolic nature of funerary rituals makes an uncritical association between observed burial features and the deceased social status (or the general characteristics of one society) a possible source of interpretive biases. Also, lacking documentary evidence, our reconstructions of funerary customs are necessarily limited to those leaving physical traces. This issue further limits a discussion of mortuary rituals based on archaeological evidence [118].

Third, funerary, isotopic, and LEH datasets only partially overlap due to the variable preservation of the skeletal and dental remains. This limited the comparability between the patterns expressed by these features, and may explain the often ambiguous or counterintuitive results of our study. Also, in scoring LEH we did not consider differences in the number of hypoplastic bands per tooth or their timing. This decision, while justified by our need to minimize possible errors or imprecise measurements, prevents a distinction between the single vs. repeated stressful episodes.

While some of the above issues are unavoidable (e.g. differential preservation of skeletal and dental remains, partial excavation of the necropolis), the collection and analysis of further data will allow refining the results of this study. Besides the mentioned expansion of the radiocarbon dated sample, and the finalization of the typological study of grave goods, future work will ideally include the analysis of ^87^Sr/^86^Sr and ^18^δO (the latter in preparation), and aDNA for the detection of mobility patterns and kinship groups, and the joint analysis of other Celtic funerary contexts from Northern Italy in order to better contextualize the biological and funerary trends observed at SV.

## Conclusion

The present is the first study exploring the association between funerary treatment, diet, and differential exposure to developmental stressors in a Celtic population from Italy. We investigate the Late Iron Age (3^rd^-1^st^ c BC) necropolis of Seminario Vescovile (Verona, Italy) applying a suite of different methods (statistical analysis of funerary, isotopic, and LEH data). Funerary data provide evidence for the presence of a nuanced differentiation of mortuary customs, which are primarily correlated to the age of the individuals. When combined with the analysis of stable isotopes and LEH, funerary data suggest at SV a lack of substantial social differences, though considering the real representativeness of the analyzed individuals, which likely depict an homogenous (and possibly low-status) segment of the original population. This study represents a preliminary step toward a better understanding of the biocultural features characterizing the Cenomani population of Italy. The future addition of supplementary data (archaeological, biochemical, and biomolecular) will increase the resolution of our analyses. A detailed bioarchaeological comparison with other Celtic contexts from Northern Italy will provide a clearer contextualization of the results from SV.

## Supporting information

S1 Fig Plan of Seminario Vescovile. **a) Overall view of the necropolis; b) Close-up of the burials** (plan by M. Bersani, by courtesy of SABAP-VR Soprintendenza archeologia, belle arti e paesaggio per le province di Verona, Rovigo e Vicenza).
(TIF)

S2 Fig Balloon plot illustrating the percentage of presence of each funerary variable by cluster. (TIF)

S3 Fig **Spatial distribution of the analyzed individuals according to sex (a), age class (b), and funerary cluster (c).** Lines show the spatial density of each group (computed with kernel density estimation).
(TIF)

S4 Fig **Spatial distribution of the analyzed individuals according to their δ**^**15**^**N (a) and δ**^**13**^**C values (b).** Shape of points describe the sex and age (adults vs. nonadults) of each individual.
(TIF)

S1 File Stable isotopes analytical methods. (DOCX)

S1 Table Complete dataset used in the study. US = Stratigraphic Unit; LEH = linear enamel hypoplasia; I1 = first incisor; I2 = second incisor; C = canine; U = upper; L = lower; l = left; r = right; LEH/Overall LEH: 0 = absent; 1 = present; NA = absence of tooth or excessive tooth wear (see text for details).Funerary variables (see S2 Table for details): 0 = absent; 1 = present; Cluster system II: CL1 plus minor clusters of CL2 (cl2,cl3,and cl4); NC2 = individuals of CL2 not included in smaller clusters.
(XLSX)

S2 Table Definition of funerary variables. (DOC)

S3 Table a): Summary statistics of number of items by age class and sex; b) Bonferroni-adjusted results of pairwise z tests for the number of ceramics between sexes. N Rings = number of rings; N Pins = number of pins; N Ceramics = number of ceramic objects; N = number; SE = standard error; Min-Max = range of values for each variable; ns: not significant.
(XLSX)

S4 Table Coefficients of the general linear models of δ^15^N and δ^13^C values with age classes and sex as predictors. a) nonadult dataset (NaI is set as reference level in the model); b) adult dataset (F and YA are set as reference levels in the model for the categories “Sex” and “Age” respectively); ns = not significant.
(DOC)

S5 Table Summary statistics of isotopic values by funerary variable. a) δ^15^N and δ^13^C values, nonadult dataset; b) δ^15^N values, adult dataset subdivided by sex and age classes; c) δ^13^C values, adult dataset subdivided by sex. Abs = Feature absent; Pres = feature present. N = number of individuals; Me = median; SE = standard error. All pairwise comparisons are not significant (p values are omitted).
(XLSX)

S6 Table **Composition of funerary clusters by age class (a) and sex (b).** *: significant results of pairwise Bonferroni-adjusted Fisher's test; not significant results are omitted.
(XLS)

S7 Table Comparison between clusters for funerary variables. a) Bonferroni-adjusted results of Fisher's tests between clusters on presence-absence of funerary variables, and b) results of post hoc Fisher's tests (only variables presenting at least one significant result are reported).
= test not possible; ns = not significant.
(XLS)
