# Supplementary figures and images for: Patterns of funerary variability, diet, and developmental stress in a Celtic population from NE Italy (3rd-1st c BC)
Source: PLoS One. 2019 Apr 17;14(4):e0214372. doi: 10.1371/journal.pone.0214372 (PMC6469778; doi:10.1371/journal.pone.0214372)

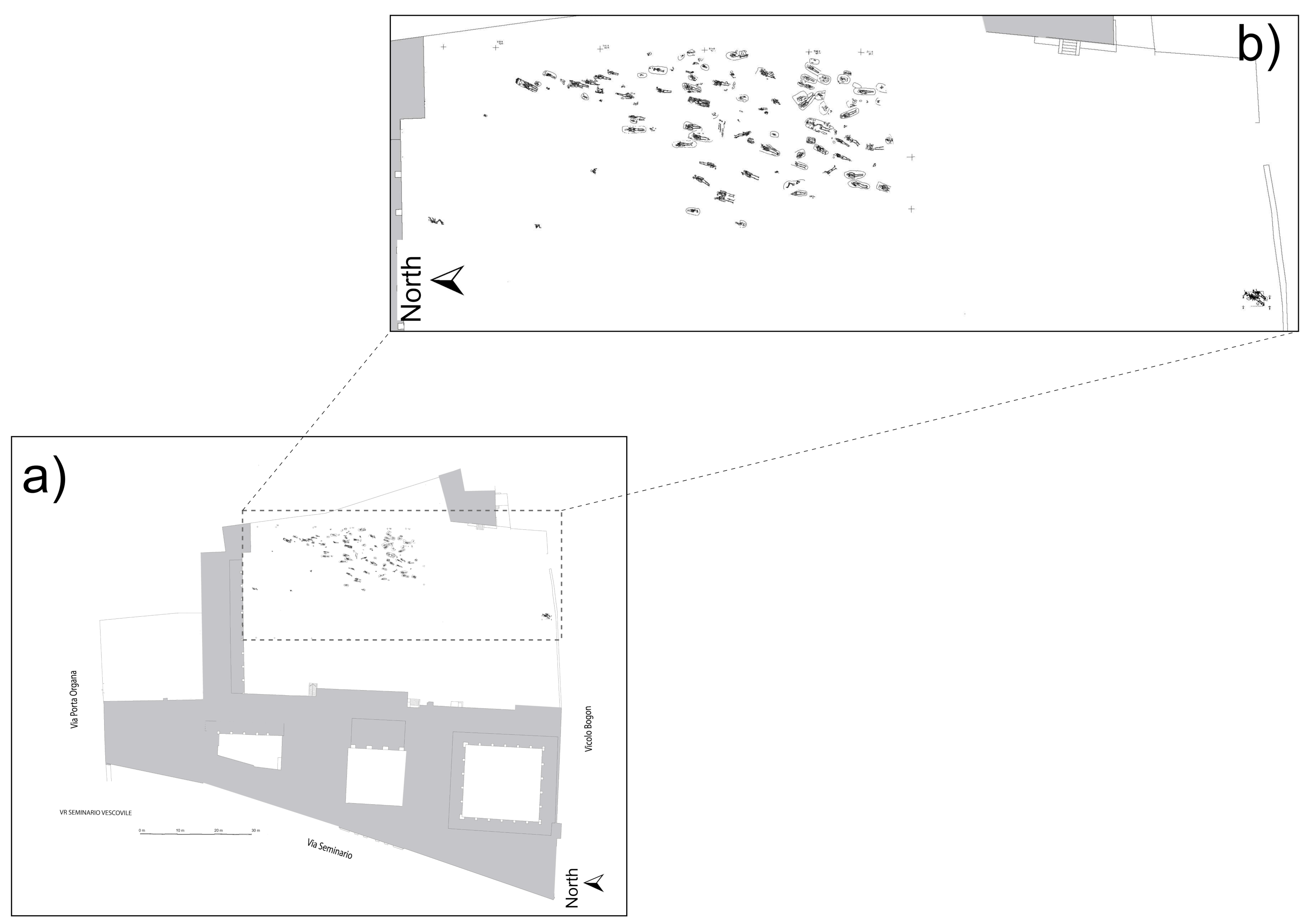

Supplement: S1 Fig — a) Overall view of the necropolis; b) Close-up of the burials (plan by M. Bersani, by courtesy of SABAP-VR Soprintendenza archeologia, belle arti e paesaggio per le province di Verona, Rovigo e Vicenza). (TIF) [file pone.0214372.s001.tif]

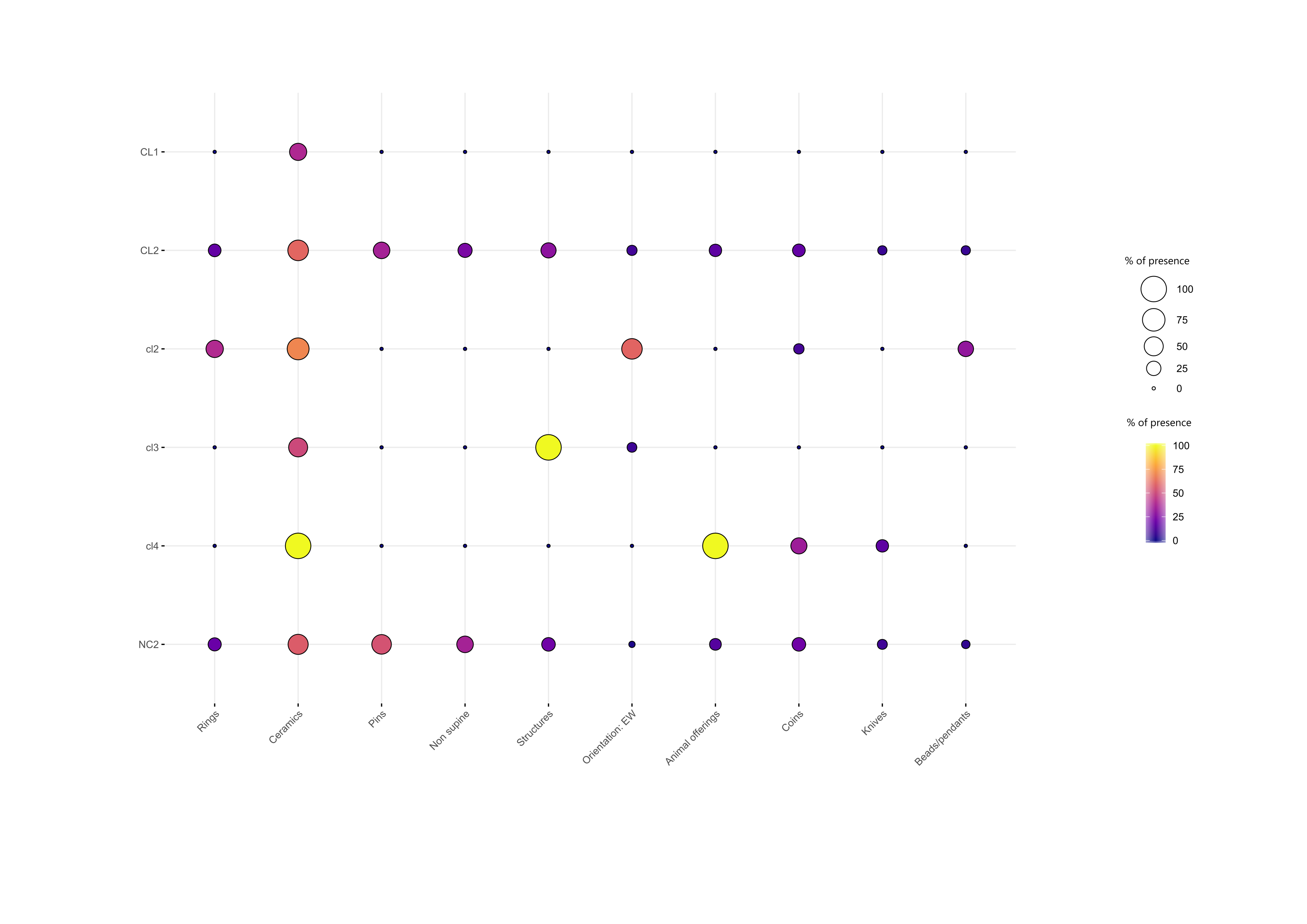

Supplement: S2 Fig — (TIF) [file pone.0214372.s002.tif]

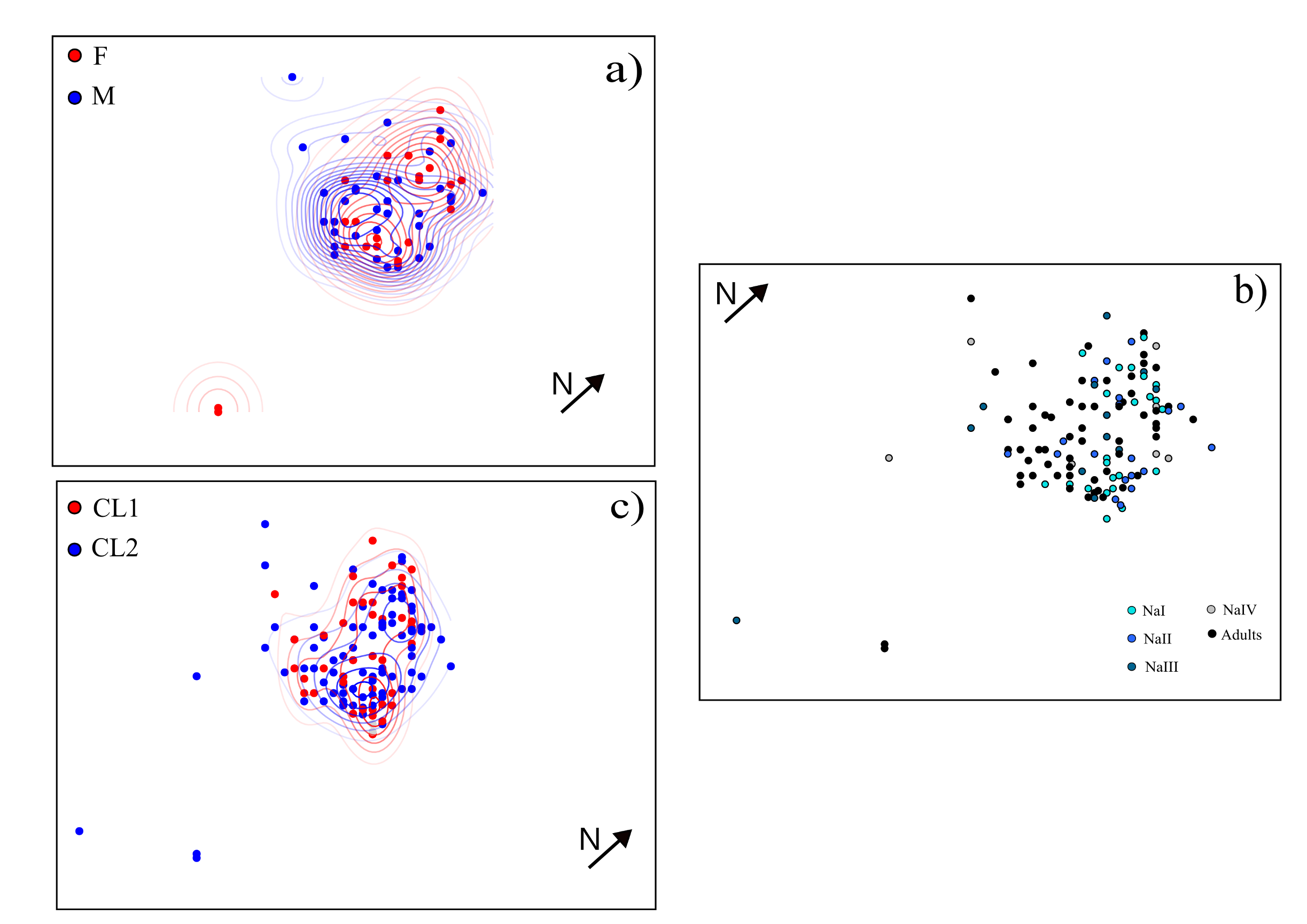

Supplement: S3 Fig — Spatial distribution of the analyzed individuals according to sex (a), age class (b), and funerary cluster (c). Lines show the spatial density of each group (computed with kernel density estimation). (TIF) [file pone.0214372.s003.tif]

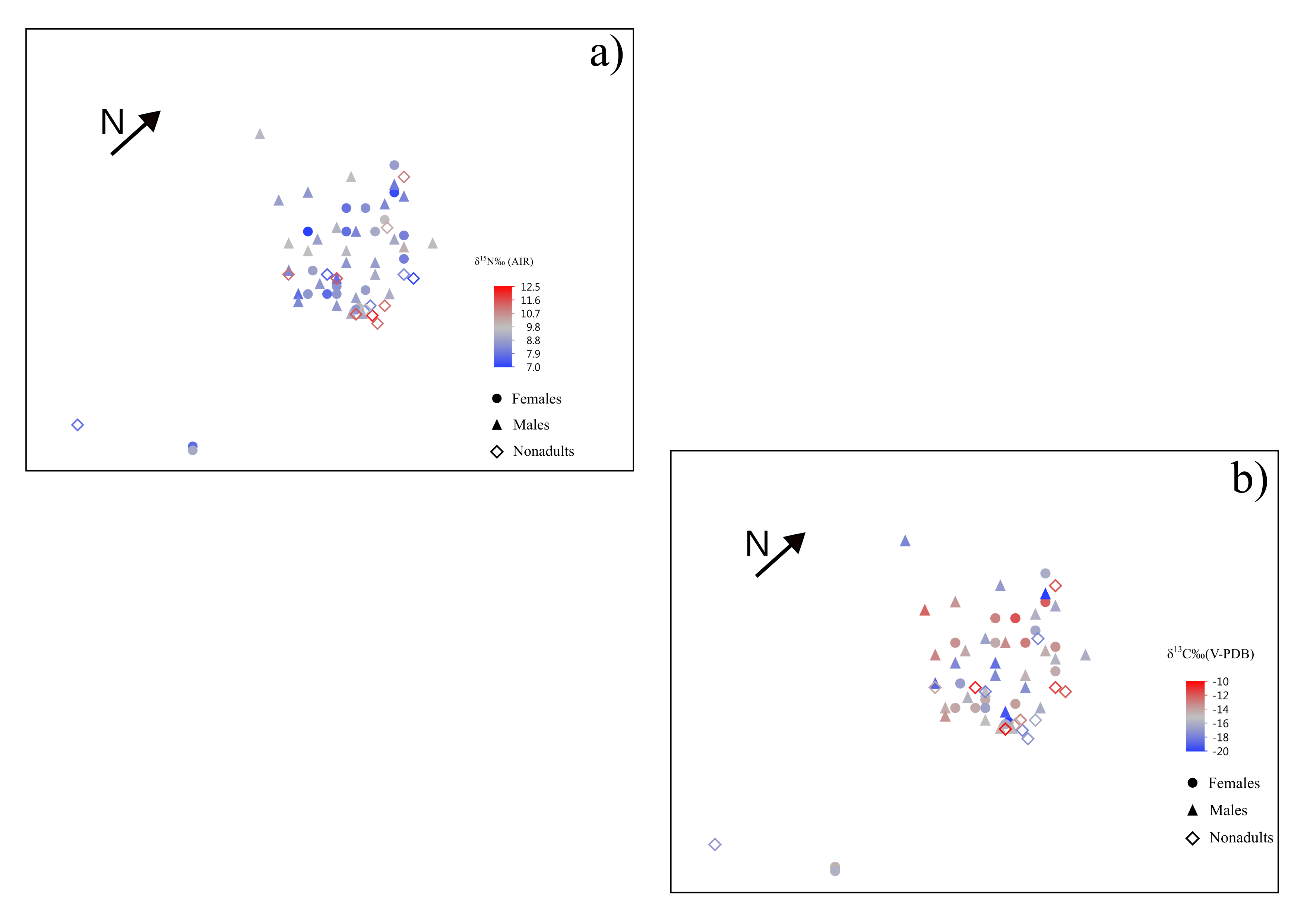

Supplement: S4 Fig — Spatial distribution of the analyzed individuals according to their δ15N (a) and δ13C values (b). Shape of points describe the sex and age (adults vs. nonadults) of each individual. (TIF) [file pone.0214372.s004.tif]
