# Supplementary material for: Patterns of funerary variability, diet, and developmental stress in a Celtic population from NE Italy (3rd-1st c BC)
Source: PLoS One. 2019 Apr 17;14(4):e0214372. doi: 10.1371/journal.pone.0214372 (PMC6469778; doi:10.1371/journal.pone.0214372)
Supplement: S1 File — (DOCX) [file pone.0214372.s005.docx]

**S1 File: Stable isotopes analytical methods**

In a first stage, collagen was extracted from the bone by a chemical procedure that ensures the removal of other organic compounds that might contaminate the sample. Rib samples were initially cleaned by scratching and then they were powdered. About 300 mg of bone powder was decalcified in 1 M HCl for 20 minutes at room temperature, eliminating phosphates, fulvic acids and other soluble acids and filtered through a MF-Millipore 5 μm filter. The insoluble residue was decanted into 0.125 M of NaOH for 20 hours at room temperature. After rinsing with Milli-Q water, the neutralized sample was filtered again (5 μm) to remove humic acids and most lipids; this residue was decanted into 10^-2^ M HCl (pH 2) solution in closed pyrex tubes, at 100° for 17 hours to solubilize the collagen. After centrifugation of the tubes for about 10 minutes, the supernatant (containing solubilized collagen) was lyophilized and analyzed for its isotopic composition. About 0.7 mg of collagen was weighed within a tin capsule per duplicate, and an elemental analyzer (Carlo Erba Model NA1500 NC) was employed for sample combustion; the N_2_ and CO_2_ obtained were separated using a chromatographic column and introduced into a mass spectrometer (Delta Plus XP) for isotope analysis. Commercial CO_2_ and N_2_ were used as the internal standards for the carbon and nitrogen isotopic analyses (see [13] for more information about the standards used). The analytical error for the δ^15^N and δ^13^C determinations was < 0.1‰.

The measurement unit was “δ”, expressed as:

δ = (R_sample_/R_standard_ – 1) *1000

where R= ^13^C/^12^C for δ^13^C values and R= ^15^N/^14^N for δ^15^N .
