# Supplementary material for: Patterns of funerary variability, diet, and developmental stress in a Celtic population from NE Italy (3rd-1st c BC)
Source: PLoS One. 2019 Apr 17;14(4):e0214372. doi: 10.1371/journal.pone.0214372 (PMC6469778; doi:10.1371/journal.pone.0214372)
Supplement: S2 Table — (DOC) [file pone.0214372.s007.doc]

**S2 Table**
